# Tissue Bioengineering with Fibrin Scaffolds and Deproteinized Bone Matrix Associated or Not with the Transoperative Laser Photobiomodulation Protocol

**DOI:** 10.3390/molecules28010407

**Published:** 2023-01-03

**Authors:** Karina Torres Pomini, Daniela Vieira Buchaim, Ana Carolina Cestari Bighetti, Abdul Latif Hamzé, Carlos Henrique Bertoni Reis, Marco Antonio Húngaro Duarte, Murilo Priori Alcalde, Benedito Barraviera, Rui Seabra Ferreira Júnior, Alexandre Teixeira de Souza, Paulo Sérgio da Silva Santos, João Paulo Galletti Pilon, Miguel Ângelo de Marchi, Dayane Maria Braz Nogueira, Cleuber Rodrigo de Souza Bueno, Wendel Cleber Soares, Rogerio Leone Buchaim

**Affiliations:** 1 Department of Biological Sciences, Bauru School of Dentistry (FOB/USP), University of São Paulo, Bauru 17012-901, Brazil; 2 Postgraduate Program in Structural and Functional Interactions in Rehabilitation, Postgraduate Department, University of Marilia (UNIMAR), Marília 17525-902, Brazil; 3 Teaching and Research Coordination of the Medical School, University Center of Adamantina (UNIFAI), Adamantina 17800-000, Brazil; 4 Medical School, University of Marilia (UNIMAR), Marília 17525-160, Brazil; 5 UNIMAR Beneficent Hospital (HBU), University of Marilia (UNIMAR), Marília 17525-160, Brazil; 6 Department of Dentistry, Endodontics and Dental Materials, Bauru School of Dentistry, University of São Paulo (FOB/USP), Bauru 17012-901, Brazil; 7 Center for the Study of Venoms and Venomous Animals (CEVAP), São Paulo State University (University Estadual Paulista, UNESP), Botucatu 18610-307, Brazil; 8 Graduate Programs in Tropical Diseases and Clinical Research, Botucatu Medical School (FMB), São Paulo State University, (UNESP–University Estadual Paulista), Botucatu 18618-687, Brazil; 9 Rector/President, University Center of Adamantina (UNIFAI), Adamantina 17800-000, Brazil; 10 Department of Surgery, Stomatology, Pathology and Radiology, Bauru School of Dentistry, University of São Paulo, Bauru 17012-901, Brazil; 11 Postgraduate Program in Speech Therapy, São Paulo State University (UNESP—University Estadual Paulista), Marília 17525-900, Brazil; 12 Coordination of the Medical School, University Center of Adamantina (UNIFAI), Adamantina 17800-000, Brazil; 13 Department of Prosthodontics and Periodontics, Bauru School of Dentistry (FOB/USP), University of São Paulo, Bauru 17012-901, Brazil; 14 Anatomy and Collective Health, Faculty of Medicine and Dentistry, University Center of Adamantina (UNIFAI), Adamantina 17800-000, Brazil; 15 Anatomy Department, Faculty of Medicine, UNINOVE University, Bauru 17011-102, Brazil; 16 Vice-Rector/President, University Center of Adamantina (UNIFAI), Adamantina 17800-000, Brazil; 17 Graduate Program in Anatomy of Domestic and Wild Animals, Faculty of Veterinary Medicine and Animal Science, University of São Paulo (FMVZ/USP), São Paulo 05508-270, Brazil

**Keywords:** biocompatible materials, bone regeneration, low-level laser therapy, photobiomodulation, bone substitutes, fibrin sealant, biopolymers, xenografts

## Abstract

Extending the range of use of the heterologous fibrin biopolymer, this pre-clinical study showed a new proportionality of its components directed to the formation of scaffold with a lower density of the resulting mesh to facilitate the infiltration of bone cells, and combined with therapy by laser photobiomodulation, in order to accelerate the repair process and decrease the morphofunctional recovery time. Thus, a transoperative protocol of laser photobiomodulation (L) was evaluated in critical bone defects filled with deproteinized bovine bone particles (P) associated with heterologous fibrin biopolymer (HF). The groups were: BC_L_ (blood clot + laser); HF; HF_L_; PHF (P+HF); PHF_L_ (P+HF+L). Microtomographically, bone volume (BV) at 14 days, was higher in the PHF and PHF_L_ groups (10.45 ± 3.31 mm^3^ and 9.94 ± 1.51 mm^3^), significantly increasing in the BC_L_, HF_L_ and PHF_L_ groups. Histologically, in all experimental groups, the defects were not reestablished either in the external cortical bone or in the epidural, occurring only in partial bone repair. At 42 days, the bone area (BA) increased in all groups, being significantly higher in the laser-treated groups. The quantification of bone collagen fibers showed that the percentage of collagen fibers in the bone tissue was similar between the groups for each experimental period, but significantly higher at 42 days (35.71 ± 6.89%) compared to 14 days (18.94 ± 6.86%). It can be concluded that the results of the present study denote potential effects of laser radiation capable of inducing functional bone regeneration, through the synergistic combination of biomaterials and the new ratio of heterologous fibrin biopolymer components (1:1:1) was able to make the resulting fibrin mesh less dense and susceptible to cellular permeability. Thus, the best fibrinogen concentration should be evaluated to find the ideal heterologous fibrin scaffold.

## 1. Introduction

The management of large bone defects has still been a challenging problem for medical and dental specialties due to the complexity of available treatments, significant morbidity and the high incidence of late complications [1]. Combined with an increasing prevalence of trauma, congenital anomalies, and degenerative diseases that can compromise the restoration of bone architecture, tissue engineering and regenerative medicine seek to develop reconstructive therapies in order to regenerate lost bone and restore its function [2,3].

In recent decades, bone substitutes have been the subject of intense investigation, with the aim of overcoming the limitations resulting from graft harvesting or using bone banks, and thus assisting and accelerating the regenerative process, repairing the lesion with new tissue with native morphofunctional characteristics [4]. Given the great diversity of commercially available biomaterials, previous studies have presented scientific evidence and predictability of clinical success in the use of xenografts of bovine origin [5].

Among the tissue engineering constructions for bone repair, the association of three-dimensional scaffolding is based on the attempt to mimic the native bone microstructure, facilitating the recruitment of osteogenic cells, in situ growth factors and promoting the synthesis of new mineralized bone matrix [6,7].

It is in this context that natural biopolymers such as fibrin derivatives have become the ideal candidate for combined employment with particulate bone grafts [8,9]. This allows for the fabrication of multifunctional scaffolds that stop bleeding by homeostatic mechanisms, increases the resistance to shear stress, and the stability of the graft in the surgical bed, which is a preponderant factor in the prevention of micromotion, and provides a longer time of cellular support during the whole process of bone repair, increasing the graft success rate [10,11].

Most of the preparations are made up of plasma blood components, which allows them to be classified according to the fibrinogen obtained, in autologous or homologous fibrin sealants. However, autologous formulations become unfeasible in severely injured patients or in unforeseen emergencies, and homologous formulations with high added value and risk of viral transmission [12].

The identification of these methodological limits spurred the team of researchers from the Center for the Study of Venoms and Venomous Animals at UNESP (CEVAP), to develop a modified version of these preparations, as an effective, safe, and affordable alternative. Thus, human fibrinogen was replaced by plasma fibrinogen from large animals, *Bubalus bubalis*, and thrombin by serine protease, extracted from the venom, *Crotalus durissus terrificus* [13].

Initially, protein concentrations of serine protease and heterologous cryoprecipitate were designed for the treatment of chronic venous ulcers, peripheral nerve repair, and an alternative to conventional sutures, with satisfactory preclinical and clinical results [14,15]. In fact, the excellent biocompatibility, controllable biodegradability, intrinsic bioactivity, and many other unique characteristics make this therapeutic formulation viable and attractive for other areas such as tissue bioengineering and regenerative medicine [13].

Thus, the improvement in research and the use of new technologies have directed the applicability of heterologous fibrin biopolymer as a three-dimensional scaffold in bone reconstruction, delivery system of biologically active molecules and support for mesenchymal stem cells [16,17,18].

In the search for improvement in the results of reconstructive surgical interventions that require tissue repair, several extra operative therapeutic modalities have been researched [19]. Among non-invasive treatments, laser photobiomodulation has been widely used in several clinical conditions in order to accelerate tissue regeneration and modulate inflammatory processes in cells with functional deficit [20,21,22].

Our team of researchers has used a laser photobiomodulation protocol in the bone defects repair process, which has achieved satisfactory and promising results [23,24,25,26,27,28]. However, a new approach to treatment frequency has been required for future research due to greater convenience, and to the need to be financially viable, allowing for the use of this technique in clinical practice. Allied to this, the scientific literature together with our previous results have pointed out the need to change the proportionality of the fibrinogen component of the heterologous fibrin biopolymer, in order to achieve a less dense three-dimensional mesh, as it is believed that this way provides a microenvironment more conducive to cell migration [21,29,30,31].

In view of these issues, this study was justified because it presented a change in the proportionality of fibrinogen in the fibrin biopolymer, with the aim of achieving the ideal characteristics of fibrin as a scaffold, providing agglutination of the particulate graft and preventing the invagination of surrounding soft tissues. Furthermore, we tested the possibility of promoting guided tissue regeneration without the use of biological barriers, such as membranes, and evaluated the use of an intraoperative protocol of laser photobiomodulation in a single session, which allows prospective clinical treatments.

Therefore, we aimed to evaluate the transoperative protocol of laser photobiomodulation in critical bone defects in the calvaria of rats, filled with deproteinized bovine bone particles associated with the new proportionality of the heterologous fibrin biopolymer components.

## 2. Results

### 2.1. X-ray Computed Microtomography (µ-CT)

**Figure 1:**
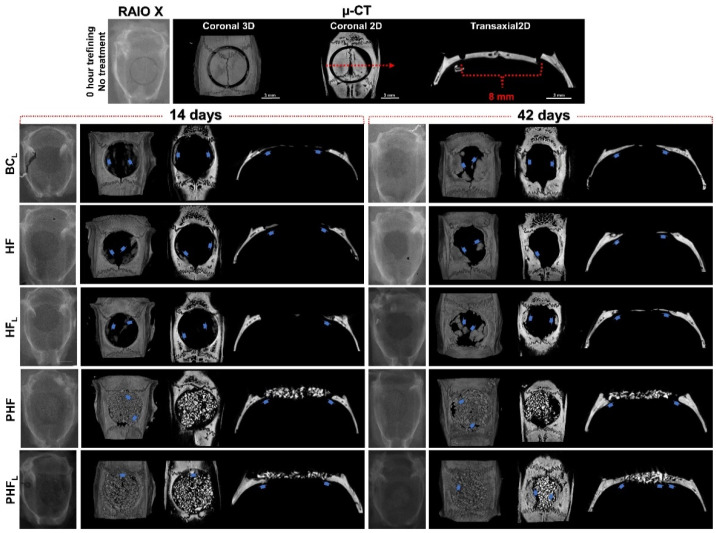
Radiographic (one-dimensional images) and computed microtomographic images (two-dimensional—top view and three-dimensional in coronal and transaxial sections) of rat calvaria bone defects at 14 and 42 days after osteotomy. (*n* = 12/group): BC_L_: defect filled by blood clot and laser photobiomodulation therapy; HF: defect filled by heterologous fibrin biopolymer; HF_L_ defect filled by heterologous fibrin biopolymer and laser photobiomodulation therapy; PHF: defect filled by deproteinized bovine bone particles incorporated into heterologous fibrin biopolymer; PHF_L_: defect filled by deproteinized bovine bone particles incorporated into heterologous fibrin biopolymer and laser photobiomodulation therapy. At 14 days, in BC_L_, HF and HF_L_, note discrete radiopacity of the defect margins vs. remaining bone suggesting moderate formation of bone tissue (blue arrow) and small bone islands in the dura mater region. In PHF and PHF_L_, the defects present a large amount of biomaterial particles and formation of centripetal bone tissue (blue arrow), which tapers towards its central region. At 42 days, bone defects were not completely restored in any experimental group. In BC_L_, HF and HF_L_, bone formation at the edge of the defect is denser and more mature, but unable to occupy the more central regions. In PHF and PHF_L_, defects with the maintenance of biomaterial particles and increased bone formation around the particles, mainly in the dura mater region, compared to the previous period. µ-CT -all scaled image size 3 mm.

The one-dimensional radiographic images and 2D and 3D reconstructed by micro computed tomography reveal the directly proportional relationship between the radiopacity of the remaining bone structures and the level of mineral present (Figure 1). Thus, the more radiopaque tones in the gray scale, closer to white, give the evaluated structures a greater degree of mineralization.

Descriptive analysis of the radiopacity of newly formed bone structures was performed in 1D, 3D (top view) and 2D (transaxial and coronal sections) planes, in order to analyze the evolution of the bone repair process in different planes and in periods of 14 and 42 days, thus verify the performance and maintenance of the biomaterial and newly formed bone architecture.

Qualitatively, in the initial period, the images of all experimental groups showed a slight increase in radiopacity contrast at the edges of the surgical wound, confirmed by their irregularities. In addition, the PHF and PHF_L_ groups showed focal areas with tiny radiopaque figures intertwining the biomaterial particles.

At 42 days, reossification was caused by the extension of bone border growths, occupying part of the defect in BC_L_, HF and HF_L_ and the spaces between particles in the PHF and PHF_L_ groups, but the calvarial defects were not completely restored. The trabecular architecture in the BC_L_, HF_L_ and PHF_L_ groups revealed more expressive formation on the dura mater and/or intimately in contact with the particles.

**Figure 2:**
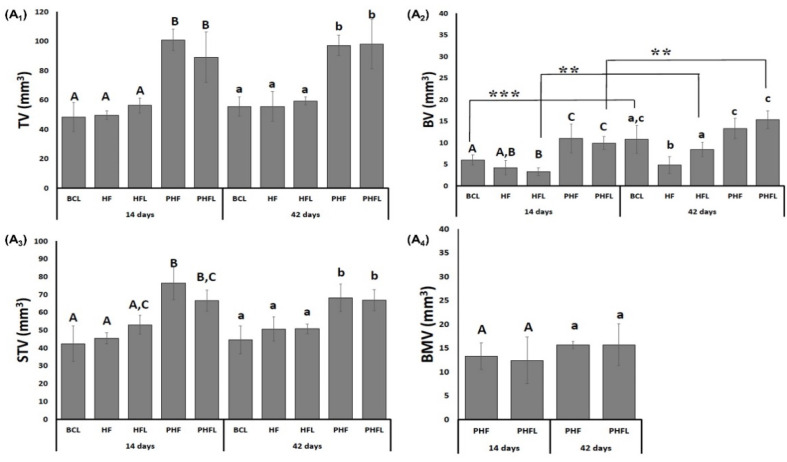
(A) Microtomographic evaluation: Mean and standard deviation graphs of the total volume evaluated, TV, (**A_1_**); bone volume, BV, (**A_2_**); soft tissue volume, STV, (**A_3_**) and the volume of BMV biomaterial particles, (**A_4_**). *n* = 12/group/period. Different uppercase (14 days, A ≠ B ≠ C) and lowercase (42 days, a ≠ b ≠ c) letters, difference between groups/period (Kolmogorov–Smirnov normality test, unpaired Student “*t*” test). Asterisk (** or ***) = significant difference between period/group; (one-way ANOVA and Tukey, *p* < 0.05).

Morphometric data of the 3D microtomographic images of the volume obtained in the CTan program are shown in Figure 2. The microtomographic images showed, in the period of 14 days, that the total volume of the region of interest (TV) was greater in the groups filled or treated with the biomaterial + biopolymer with or without laser (PHF/PHF_L_, mean of 94.9 ± 12.22 mm^3^) in comparison to those filled with clot or biopolymer with and without laser (BC_L_/HF/HF_L_, mean of 51.45 ± 6.01 mm^3^), remaining constant at 42 days (*p* < 0.05).

Regarding the bone volume (BV), at 14 days it was significantly higher (*p* < 0.05) in the groups filled with biomaterial + biopolymer (PHF, 10.45 ± 3.31 mm^3^) and biomaterial + biopolymer and laser (PHF_L_, 9.94 ± 1.51 mm^3^) and lower in the groups filled with clot or biopolymer with or without laser (BC_L_/HF/HF_L_, mean 4.51 ± 1.25 mm^3^). In the percentage data at 14 days, PHF had 126% greater bone volume compared to BC_L_/HF/HF_L_ groups.

At 42 days, BV significantly increased in the groups that received BC_L_ (10.78 ± 3.27 mm^3^), HF_L_ (8.44 ± 1.68 mm^3^) and PHF_L_ (15.35 ± 2.09 mm^3^) laser application and did not show significant differences in the HF groups (4.83 ± 1.17 mm^3^) and PHF (13.32 ± 2.33 mm^3^). Percentagewise, the PHF_L_ group showed 23% greater than BC_L_ and 57% greater than HF_L_.

Regarding soft tissue volume (STV), at 14 days, the presence of biomaterials in the PHF and PHF_L_ groups gave a higher mean (71.58 ± 7.68 mm^3^, *p* < 0.05) in relation to the BC_L_, HF and HF_L_ groups (mean of 46.93 ± 6.09 mm^3^), persisting until the end of the experiment.

In the groups treated with biomaterial with and without laser, the volume of biomaterial (BMV) was similar, with a mean of 14.25 ± 3.22 mm^3^, and with no changes at 42 days.

### 2.2. Histomorphological Analysis

**Figure 3:**
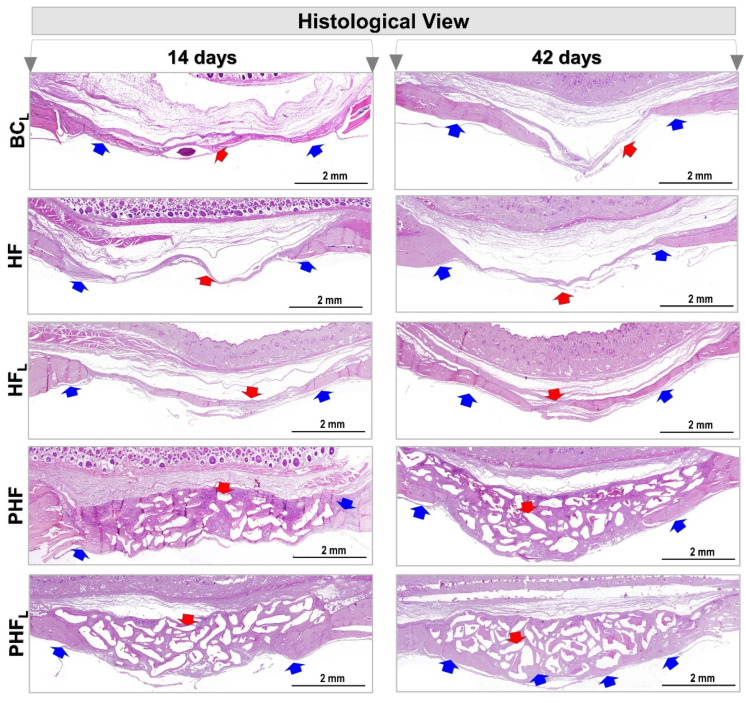
Panoramic histological view of cranial defects created in animals at 14 and 42 days. (*n* = 12/group): BC_L_, HF, HF_L_, PHF and PHF_L_ groups. Panoramic aspects of the calvaria showing height and conformation compromising the type of treatment. At 14 days, in BC_L_, HF and HF_L_, areas of defects filled predominantly by connective tissue (red arrow), were observed mainly in BC_L_, with areas of neoformed bone tissue (blue arrow) on the edges of the defect, and by rich connective tissue in cells and vascularized in the most central region. In the PHF and PHF_L_ groups, centripetal bone growth at the margins of the surgical area, presence of connective tissue permeating the biomaterial particles (red arrow). At 42 days, bone growth remained restricted to the margins of the defect, and wound closure was predominantly by connective scar tissue and/or biomaterial. (HE original magnification 4×; bar = 2 mm).

All experimental groups presented centripetal bone deposition throughout the course, that is, the new bone tissue was made from the surgical edges and the dura mater surface towards the center of the defect. However, this growth was not regular along the entire circumference (Figure 3).

At 14 days, in the BC_L_, HF and HF_L_ groups, neoformation of primary bone tissue was observed, with bone beams randomly distributed and richly cellularized by newly trapped osteocytes and covered on their surfaces by osteoblasts. There was abundant granulation tissue, containing diffuse collagen fibers, macrophages, vascular neoformation, fibroblasts and little extracellular matrix filling the central region of the wound.

Since, in the PHF and PHF_L_ groups, the defects were filled by the biomaterial particles of different sizes, and reactive connective tissue, which are prominent in PHF. Comparatively, bone formation on the dura was more expressive in PHF_L_ and fine bone trabeculae permeating the particles, with richly cellularized connective tissue also being observed.

At 42 days, an increase in bone repair was observed in all experimental groups, but none of the defects fully restored the lost bone structure, being occupied by scar connective tissue and/or biomaterial particles.

Defects in the BC_L_ group showed integument collapse into the defect, compromising the restoration of local bone thickness of approximately 1 mm of native bone plate. However, in the HF and HF_L_ groups, the area of the surgical cavity remained without the presence of epithelial tissue (Figure 3).

Areas of bone formation and renewal were observed in BC_L_ and HF_L_, demonstrated by the presence of young bone, trabecular arrangement, unorganized bone structure, and compact, lamellar, and dense bone (see Figure 3), unlike the HF group which is predominantly with osteoid matrix.

In the other groups, PHF and PHF_L_, the new bone tissue formed remained restricted to the margins of the receptor bed, and the ossification locus with dense and lamellar arrangement, intimately adsorbed to the biomaterial particles were observed, mainly in PHF_L_ and surrounded by collagen fibers and resorptive cells in depressions of the excavated matrix. Although there were no significant changes in the amount of biomaterial in the two cases, some areas of discontinuity were exhibited and filled by connective scar tissue.

**Figure 4:**
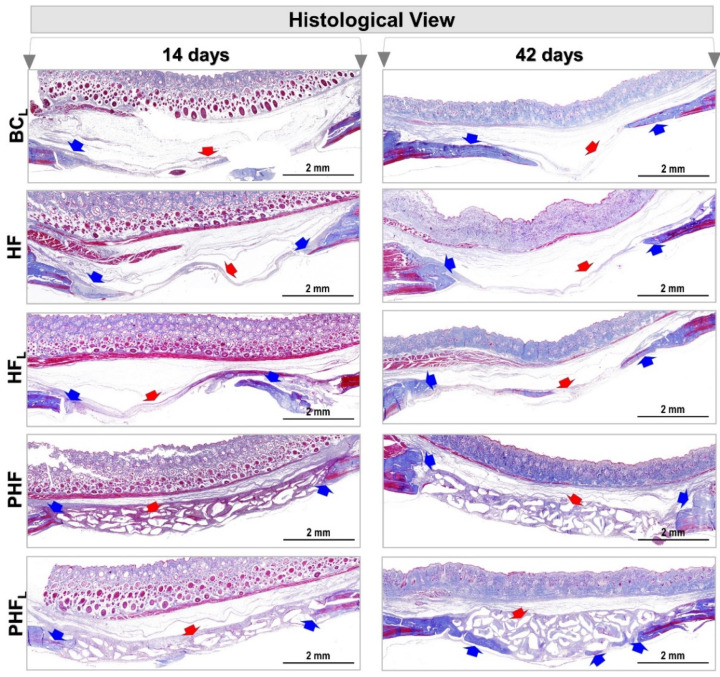
Photomicrographs of calvaria defects in rats at 14 and 42 days. *n* = 12/group): BC_L,_ HF, HF_L,_ PHF and PHF_L_ groups. Panoramic aspects of the calvaria showing height and conformation compromising the type of treatment. Blue arrow (new bone tissue formed) and red arrow (fibrous connective tissue and/or biomaterial particles), (MT original magnification 4×; bar = 2 mm).

Staining with Masson’s trichrome (MT) in the BC_L_ and HF groups showed a central area of the defects connected by a thin layer of light blue tissue corresponding to fibrous connective tissue throughout the experimental period (Figure 4).

On the other hand, the other groups showed transition from light blue to dense blue areas, interconnected with immature extracellular matrix locus, central and/or adjacent to the edges of the defect, densely stained with red, and in PHF and PHF_L_, permeating the particles of the biomaterial. The dense blue color has high specificity with collagen staining; thus, the descriptive results show that the HF_L_ group has a greater expressiveness of collagen within the defect area compared to the others.

**Figure 5:**
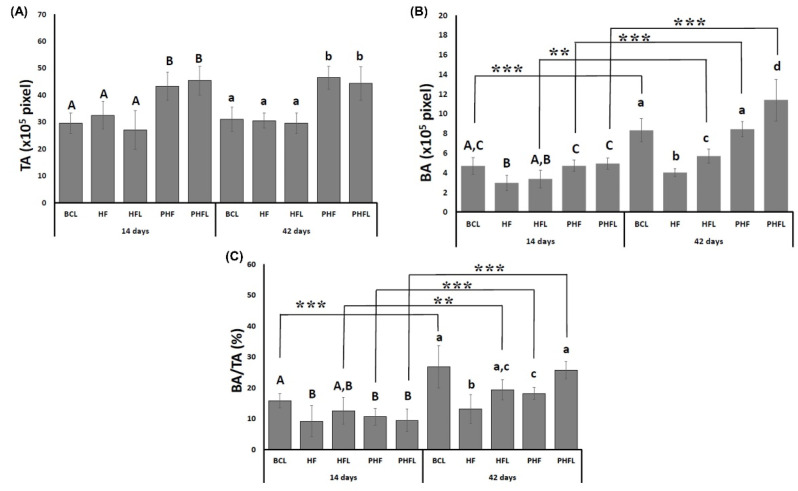
Histomorphometric evaluation: Graphs of the mean and standard deviation of the total area—TA (pixels), (**A**); bone area (pixels)—BA, (**B**); bone area/total area—BA/TA (%) (**C**); *n* = 12 group/period. Different uppercase (14 days, A ≠ B ≠ C) and lowercase (42 days, a ≠ b ≠ c ≠ d) letters, difference between groups/period (Kolmogorov–Smirnov normality test, unpaired Student “*t*” test). Asterisk (** or ***) = significant difference between period/group; (one-way ANOVA and Tukey, *p* < 0.05).

As observed in the volumetric evaluations by micro-CT, the total area, TA, at 14 days was higher in defects filled with the biomaterial, PHF and PHF_L_ (mean grafted area of 44.31 ± 5.33 × 10^5^ pixels) in relation to those without BC_L_ biomaterial, HF and HF_L_ (bone defect area, mean of 29.7 ± 1.7 × 10^5^ pixels) and remained similar at 42 days (Figure 5A).

Regarding BA, at 14 days, it was significantly higher in the BC_L_, PHF and PHF_L_ groups (mean of 4.77 ± 0.65 × 10^5^ pixels) compared to HF and HF_L_ (mean of 3.17 ± 0.83 × 10^5^ pixels). At 42 days, BA increased significantly in all groups except HF and was significantly higher in laser-treated groups. The evaluation of the percentage of newly formed bone tissue in the defect, (BA%, showed that bone formation was small until 42 days, not exceeding 30% of the defect or grafted area (Figure 5B).

In TA, it was higher in the groups treated with biomaterial, the percentage values within the evaluated area show variations in relation to those obtained for the BA. At 14 days, the BA/TA in the BC_L_ group (15.83 ± 2.34%) was similar to the HF_L_ (12.48 ± 4.35%) and significantly higher compared to the other HF groups (9.14 ± 5.01%), PHF (10.63 ± 2.73) and PHF_L_ (9.44 ± 3.65%). At 42 days, BA/TA increased significantly in the BC_L_ laser-treated groups (26.78 ± 6.80%), PHF_L_ (25.71 ± 2.79%), HF_L_ (19.3 ± 1.92%), followed by PHF (18.13 ± 1.95%). The lowest BA/TA values were observed in the HF group (13.18 ± 4.65%) (Figure 5C).

### 2.3. Birefringence Analysis of Collagen Fibers in Bone Neoformation

**Figure 6:**
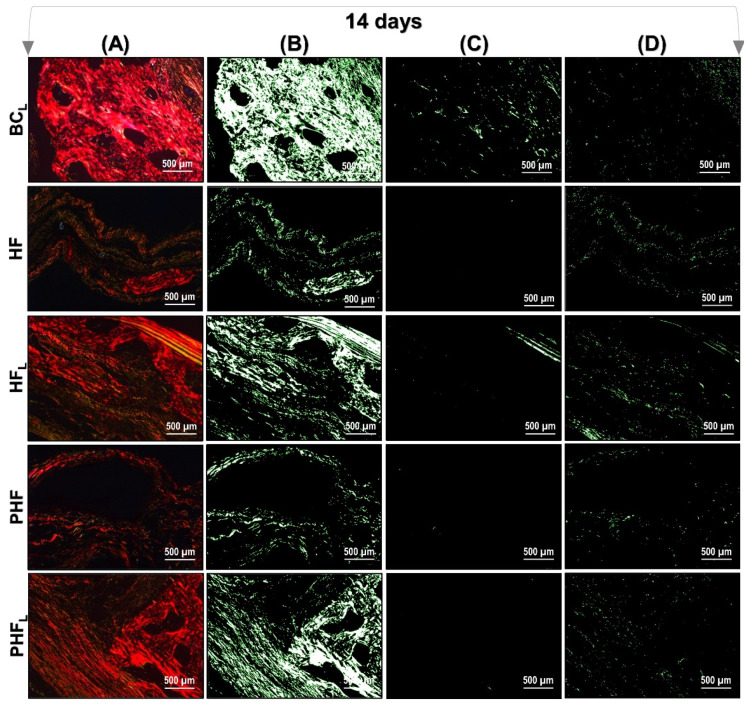
Images of Picrosirius-red stained sections observed by polarized light microscopy after 14 days. *n* = 12/group): BCL, HF, HFL, PHF and PHFL groups. (**A**) Polarized image showing the deposition of birefringent collagen (arrows); (**B**) Regions marked in red; (**C**) Regions marked in yellow corresponding to polarizing collagen deposition; (**D**) Regions marked in green. The yellowish–green birefringence of collagen fibers corresponds to thin bundles with diverse and disorganized arrangements, and reddish corresponds to dense and organized bundles, Picrosirius-red under polarization, 40× objective. Bars = 500 µm.

**Figure 7:**
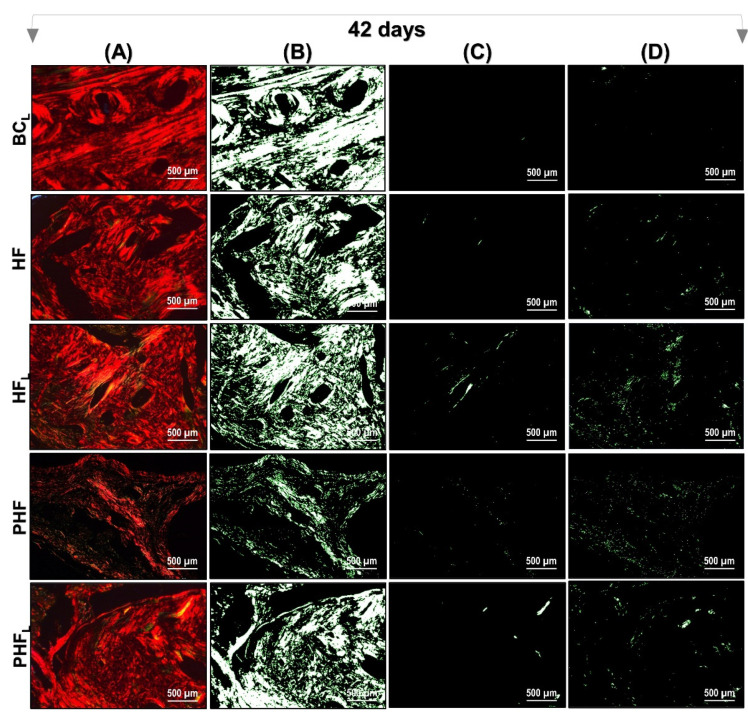
Images of Picrosirius-red stained sections observed by polarized light microscopy after 42 days. *n* = 12/group): BCL, HF, HFL, PHF and PHFL groups. (**A**) Polarized image showing the deposition of birefringent collagen (arrows); (**B**) Regions marked in red; (**C**) Regions marked in yellow corresponding to polarizing collagen deposition; (**D**) Regions marked in green. The yellowish–green birefringence of collagen fibers corresponds to thin bundles with diverse and disorganized arrangements, and reddish corresponds to dense and organized bundles, Picrosirius-red under polarization, 40× objective. Bars = 500 µm.

Figure 6 and Figure 7 show a representation of the images generated in polarization microscopy. Through qualitative analysis, it is possible to observe the variation in brightness, according to the distribution pattern of collagen fibers and to estimate the level of bone maturation of the newly formed bone in the selected periods.

After light polarization, the primary bone tissue was recognized by its random and disorganized fibrillar pattern, usually with polarization colors ranging from green/yellow and the lamellar-compact bone has a red–orange colour depending on the fiber width. In the initial period (14 days) of bone deposition, in all experimental groups, yellow–green fibers were predominantly detected filling the entire length of the receptor bed connecting the surgical edges. However, the HF_L_ group presented yellow–green birefringence locus in central areas (see Figure 6).

At 42 days, all groups remained with a reddish birefringence domain, transitioning slightly to yellowish–greenish birefringence. However, it was noted that there is qualitative evidence in the appearance of the organization of collagen fibers, indicating that the groups treated with lasers are in better conditions of structural organization (see Figure 7).

**Figure 8:**
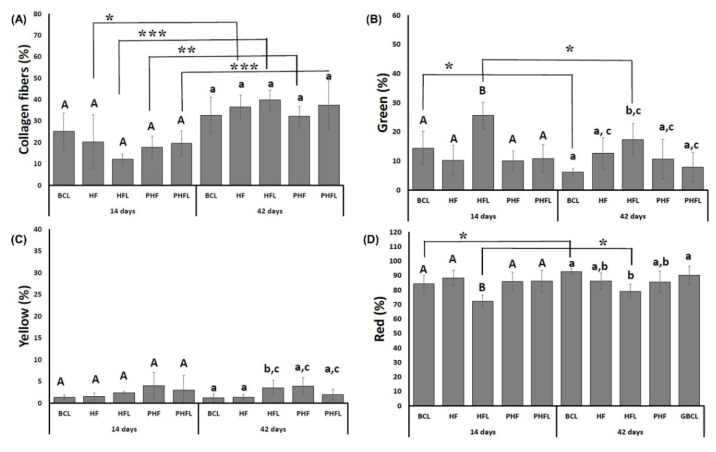
Graphs of the mean and standard deviation of the total percentage of birefringence of collagen fibers (**A**); green birefringence of collagen fibers (**B**); yellow birefringence of collagen fibers (**C**); red birefringence of collagen fibers (**D**). *n* = 12 group/period. Different uppercase (14 days, A ≠ B) and lowercase (42 days, a ≠ b ≠ c) letters, difference between groups/period (Kolmogorov–Smirnov normality test, unpaired Student “*t*” test). Asterisk (* or ** or ***) = significant difference between period/group; (one-way ANOVA and Tukey, *p* < 0.05).

The quantification of collagen fibers stained by the Picrosirius-red technique (PRS) showed that the percentage of collagen fibers in the bone tissue was similar between the groups for each experimental period, but significantly higher at 42 days (35.71 ± 6.89%) than at 14 days (18.94 ± 6.86%) (Figure 8).

Regarding the thickness of collagen fibers both at 14 and 42 days, the percentage of red/thicker fibers (mean of 85.03 ± 5.54%), followed by green/less thick (mean of 12.57 ± 4.78%) and yellow fibers (mean of 2.4 ± 1.45%). At 42 days, the highest percentage of green fibers was observed in the HF_L_ groups (25.59 ± 4.59%) in relation to the other groups, resulting in the lowest percentage of red fibers (71.11 ± 4.3%).

## 3. Discussion

The bone’s capacity for self-renewal and remodeling in response to pathophysiological changes the imposition of biomechanical stress and in situations of fractures is remarkable, which allows the tissue to return to its native state without leaving a scar [32]. However, this specificity of bone repair becomes limited in conditions of extensive tissue involvement, requiring clinical and surgical interventions to realign the repair process [33].

In view of the importance of bone reconstructions in order to restore local microarchitecture, as well as molecular mechanisms, in a way that facilitates the cellular infiltration necessary in bone repair; in addition, advances in tissue engineering approaches have driven the search for the association of materials, that is, of tissue construction, that provide a permissive environment for bone healing to occur [34,35]. Therefore, the present study focused on the development of a new tissue construction and proposed a unique protocol of laser photobiomodulation therapy, in order to radically reduce application sessions, and generate perspectives in the expansion of translational use.

A brief description of the dosimetric parameters used in this study are described in Table 1:

Considering that this study aimed to investigate the effect of laser on critical bone defects in the calvaria of rats filled with the new three-dimensional construction of the heterologous fibrin biopolymer associated with deproteinized bovine matrix, the results denote potential effects of laser radiation capable of inducing a functional bone regeneration.

Before proceeding with the descriptions of the analyses, it is important to justify the non-performance of the group with defects filled by blood clot (BC) in this study, as we prioritize the principle of the three Rs, as proposed by reduction, substitution, and refinement in the use of animals, with the objective of constantly promoting a balance between scientific progress and animal welfare [45,46,47]. Likewise, because such a group has already been carried out in our previous studies with the same methodology, animal model, and experimental period (volume density of the newly formed bone, mean 7.06 ± 0.49 and bone volume, mean 5.20 ± 1.02) [21].

The physicochemical characteristics of the particulate biomaterial used in this study (Commercial Samples—Bio-Oss^TM^, Geistlich Pharma AG, Wolhusen, Switzerland) are previously reported in the literature: total intruded volume (0.546 cm^3^/g), mode of intraparticle pores (0.03 µm), total porosity (63.5%), and intraparticle porosity (51%—taken as the percentage of the particles internal pores (<1 µm), relative to the total), particle size (250–1000 µm—Size range reported by producers) [48]. Studies recommend that the ideal diameter of the particles be between 200 and 350 μm, which ensures in this experiment that such properties provided vascularization, fluid diffusion and cellular recruitment to the defect produced [49]. As for the proliferation of mesenchymal cells in the fibrin biopolymer scaffold, it was demonstrated in the studies by Gasparotto et al. (2014) [50], in which, through investigations of flow cytometry, light and electron microscopy, he proved the satisfactory plasticity and excellent capacity of interaction of the three-dimensional matrix and the mesenchymal stem cells (MSCs) [50].

The possible mechanisms of cell proliferation and how laser-treated scaffolds increase the effect of cell proliferation can be explained by the cells that aresurrounding the wound, intrinsically related to the bone regeneration process, bind directly to fibrin, through platelet surface receptors, the integrins, providing the adsorption of these ligands. Previous studies suggest that electromagnetic laser radiation increases the expression of these cell adhesion molecules, increasing the density of binding sites and concomitantly cell infiltration and proliferation [51]. In addition, another factor to be elucidated, is the action of the laser on fibroblasts since these cells penetrate the wound in order to synthesize type I collagen. Thus, the greater deposition and organization of collagen fibril bundles generated by radiation modulation provides a synergistic effect as cellular support of the three-dimensional fibrin network at the lesion site [51].

On the other hand, the laser activates NF-ĸB ligand receptors (RANKL), inhibiting osteoclast formation during the differentiation process. This fact substantially reduces the expression of the plasminogen receptor present in these precursor cells of the phagocytic mononuclear system, delaying fibrinolysis [52].

Microtomographically, two- and three-dimensional reconstructions made it possible to investigate bone modeling, as well as obtaining volumetric measurements within bone defects [53]. Thus, at 14 days, the central hypodensity observed in BC_L_ corresponds to the region occupied by granulation tissue and osteoid matrix (see Figure 1). The presence of mineralized bone trabeculae under the dura mater was evident by the hyperdensity of notable structures in this period, but without closure of the surgical wound by mineralized tissue at 42 days, which can be characterized as a defect of critical size [54].

Since, in the same period, the defects filled with biomaterials, PHF and PHF_L_, in all imaging planes, denote difficulty in distinguishing the new bone tissue, strongly interwoven with the particles, due to their isorradiographic density and the remaining bone [55]. Additionally, considering thresholding, the strong contact between the surface of the xenogenic particle and the native bone, may have contributed to the lower selectivity of the different fractions based on gray values [56].

At 42 days, in all experimental groups, the mineralized bone tissue was restricted to the edges of the defects, with some sparse mineralization nuclei in the center of the surgical area and/or intertwining the biomaterial particles. These findings are consistent with the studies by Chen et al. (2021) [56], which attributed to the regenerated bone restricted the margins of the defect, exclusively to bone progenitor cells migrated from adjacent native bone tissues. It is also believed, due to the presence of xenogenic biomaterial particles, even at 42 days, it may have selectively hindered cell infiltration and, consequently, the diffusion of paracrine factors and nutrients to the center of the defect, justifying the absence of restoration of the calvarial bone plate.

As an additional analysis, 3D images of the calvaria were obtained in microtomography for the comparative measurement between the groups regarding bone volume, particulate material, and soft tissue. At 14 days, a significant difference was observed in the percentage of total tissue volume (TV) present in the region of interest (ROI) between PHF and PHF_L_ vs. BC_L_, HF and HF_L_ (means 94.9 ± 12.22 mm^3^ vs. 51.45 ± 6.01 mm^3^), inherent to the volume occupied by the filling materials vs. bone block removed, respectively.

As the TV was higher in PHF and PHF_L_ and bone formation was restricted to the edges of the defect, the total soft tissue volume, STV, was higher in these groups with a mean of 71.58 ± 7.68 mm^3^ in relation to BC_L_, HF and HF_L_, mean of 46.93 ± 6.09 mm^3^, in the analyzed periods, data consistent with the studies of Lappalainen et al. (2016) [57]. Regarding the variable BV, at 14 days, it showed the highest means in PHF and PHF_L_ compared to the others (10.45 ± 3.31 and 9.94 ± 1.51 vs. 4.51 ± 1.25, *p* < 0.05). According to preliminary evidence, the granular configuration of the xenogenic biomaterial associated with the three-dimensional conformation of the fibrin mesh may have contributed to a greater biomimetic surface area, initially facilitating microvascular penetration, adsorption of bioactive molecules and cellularization of autologous tissue [58].

Additionally, the explanation for such data can be elucidated by other studies that point out that the presence of the graft material prevents the infiltration of supracalvarial tissues in the surgical area, and consequently the competition for cell adhesion between osteoblasts and fibroblasts, facilitating the growth of osteoprogenitor cells [59,60]. At 42 days, the mean bone volume, BV, showed a significant increase in the BC_L_, HF_L_ and PHF_L_ groups, which can be explained by the considerable decrease in the severity of the inflammatory response in the initial periods, through the inhibitory activity of the laser energy in the release of pro-inflammatory chemical mediators such as TNF-α, IL-1β and IL-6 [61].

As for the averages of BMV, it was observed that there was no change (PHF and PHF_L_, *p* ≥ 0.05) during the entire experimental period. Considering the compatible time for replacement of biomaterial particles by bone tissue, there is still a lack of consensus in the literature. Some authors report that xenogenic particles of Bio-Oss^TM^ (Geistlich Pharma AG, Wolhusen, Switzerland) are not bioreabsorbed, being incorporated into the new bone tissue formed inside the bone defects [62,63].

On the contrary, other studies reveal that after months of repair, osteoclastic activity is observed on the surface of the particles, decreasing the pH in the microenvironment, which provides the breakdown of hydroxyapatite from the bovine bone matrix. This fact suggests a remodeling of the bone/particle set, which, in a way, characterizes a slow process of bioresorption and replacement [64,65]. According to the histological results (Figure 3 and Figure 4), a sequence of similar steps is observed in the BC_L_, HF and HF_L_ groups, with the complete filling of the defects by a thin layer of fibrous connective tissue at 42 days. Such evidence is compatible with the chronology of bone repair of extensive defects in rat calvaria, described previously and confirmed by the microtomography images, reported previously [21,22,35,66,67].

Thus, at the end of the period, the morphological characteristics of the original diploe, in all experimental groups, were not reestablished either in the external cortical bone or in the epidural, with only partial bone repair of defects, similarly observed in the Kretlow et al. (2010) [68] and Pires et al. (2021) [69] studies. Thus, the histomorphometric data were compatible with the microtomographic data in volume/mm^3^ and area/mm^2^, with TV and BA values significantly higher in the groups treated with lasers at 42 days.

In fact, a single intraoperative laser session with a wavelength of 808 nm and an energy dose of 6 J for 60 s/point, already established in the scientific literature [70,71,72], as demonstrated by the effectiveness in the first stage of the procedure which is the Repair process, as greater proliferation and cell division occur in this period, providing an increase in the volume of the newly formed bone. In addition, the laser stimulates the expression of active molecules intrinsically related to the reduction of exudate by the activation of macrophage cells in situ, as could be observed discrete inflammatory infiltrate at 14 days [73].

Following these findings, it is also believed that laser radiation significantly increases mast cell degranulation in the first 24 h after tissue injury, promoting a transient amplification of the acute inflammatory response, followed by a substantial reduction in neutrophil concentration. This result leads to an indication of the crucial role of laser in bone repair in the early stages, as it modulates the inflammatory response to accelerate the acute phase and, thus, culminate in the anticipation of the chronification of the process and, consequently, promote bone formation [74].

Expanding on histological investigations, the analysis of the structural organization of collagen fibers in the microarchitecture of the new bone tissue formed, by means of Picrosirius-red staining, confirmed the intrinsic relationship between the different shades of birefringence with the quantity and thickness of the aligned filaments. Thus, the birefringence tone emitted by collagen fibers can vary from green, indicating thinner and more dispersed fibers, changing from yellow to red, indicating gradually thicker and organized fibers with a greater degree of compaction (see Figure 6 and Figure 7) [75].

Therefore, it was found that the three birefringence tones were seen in all experimental groups, with a similar pattern among them in terms of quantity, organization, and alignment of collagen fibrils. However, at 42 days, there was a greater deposition of collagen matrix versus the previous period (18.94 ± 6.86%, 14 days vs. 35.71 ± 6.89%, 42 days), with a predominance of thicker fibers/red (mean 85.03 ± 5.54%).

Taking these results into account, it is suggested that a gene upregulation of type 1 collagen, COL-I, has occurred, similar between the groups at the end of 42 days, which points to a greater presence of fibroblasts and active osteoblasts in locus, triggering in a better organization of the primary trabecular bone and consequently of the mature bone. In view of the results presented, it is undeniable that the new proportionality of the fibrinogen component of the heterologous fibrin biopolymer played a fundamental role in hemostasis during the surgical procedure, and as an agglutinating agent for graft particles, contributing to greater wound stability [76].

It is also worth mentioning that the present study proposed a new proportionality of the fibrinogen component of the heterologous fibrin biopolymer, in order to reduce the stiffness and high density of the fibrin mesh, as seen in our previous studies [25,30]. Thus, it is believed that it enables a tissue construction with pore morphology compatible with the native bone matrix, enabling the incorporation and adsorption of bioactive molecules and cells.

Finally, the placement of the particulate xenograft in the calvarial defect, at 14 days, provided support for cell fixation, consequently presenting a higher bone percentage (BA/TA%). However, at 42 days, it was expected that defects filled with tissue construction treated with laser radiation, PHF_L_, would present statistically significant higher means, but this was not demonstrated. The suggestion for this finding lies in the fact that the particulate material may have exerted a shielding action against electromagnetic radiation to the cells, inhibiting the regenerative action of the laser treatment, as observed in the studies by Luca et al. (2020) [77].

In view of the above, it is believed that the set of evidence raised in the present work allowed us to obtain new and relevant information, which may contribute to the understanding of the effects of laser on bone repair treated with different bone grafts associated with fibrin biopolymer with alteration in the proportionality of fibrinogen.

Although we have noticed differences between the experimental groups in the investigations carried out, we consider future molecular and immunohistochemical analyzes, which can serve to compare the expression of cytokines intrinsically related to the inflammatory process, and to cell recruitment during bone repair.

## 4. Materials and Methods

### 4.1. Deproteinized Bovine Bone Particles

Bio-Oss™ (Geistlich Pharma AG, Wolhusen, Switzerland) is an inorganic matrix of sterilized bovine cortical bone consisting of a structure and calcium-phosphorus ratio similar to human bone hydroxyapatite (Ministry of Health Registry Brazil No. 806.969.30002; granules 0.25–1 mm; lot 8160089) (Figure S1, Supplementary Materials).

### 4.2. Heterologous Fibrin Biopolymer

The fibrin biopolymer, formerly called fibrin sealant derived from snake venom, was provided by the Center for the Study of Venoms and Venomous Animals (CEVAP) at São Paulo State University (UNESP), Botucatu, São Paulo, Brazil, whose components and application formula is in accordance with patent number BR 102014011432-7 issued on 6 July 2022 by the National Institute of Industrial Property of Brazil (INPI) [78]. It underwent a phase I/II clinical trial, which proved its safety for therapeutic use in humans, standing out as a promising therapeutic potential [79].

The biopolymer is composed of three solutions, previously thawed, mixed, and homogenized before the application. Fraction 1 is a thrombin-like enzyme extracted from *Crotalus durissus terrificus* venom, the diluent comprises calcium chloride and fraction 2 is a cryoprecipitate rich in fibrinogen produced from *Bubalus bubalis* blood. The proportion and the amount were used (1:1:1) and readjusted according to the research needs [11,12,14,80]. (Figure S1, Supplementary Materials).

### 4.3. Selection and Maintenance of Animals

**Figure 9:**
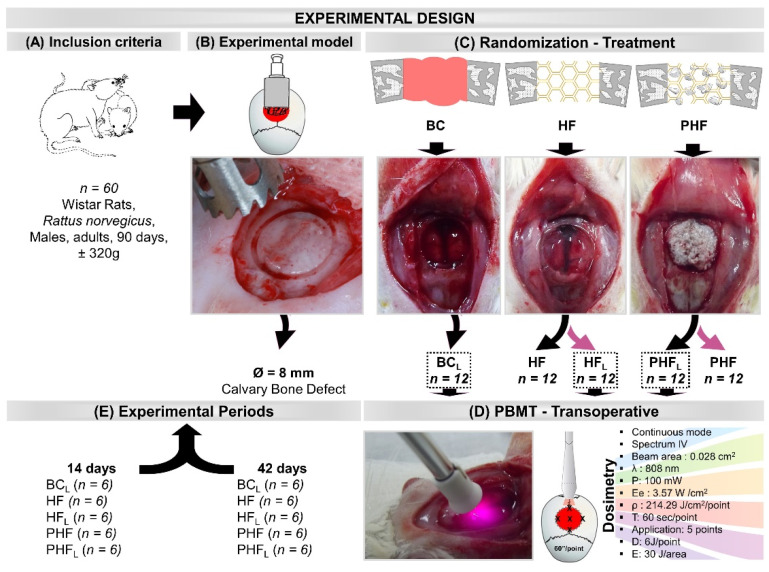
Illustrations and images of the experimental design. (**A**) Inclusion criteria: 60 rats, adults, Rattus norvegicus, Wistar hannover strain, males, age 90 days, body mass of approximately 320 g. (**B**) Experimental model: Bone defect in the center of the parietal bones with the aid of a trephine drill, 8 mm in diameter. (**C**) Randomization (*n* = 12/group) and Treatment: BC_L_—defect filled by blood clot and laser photobiomodulation therapy; HF—defect filled by heterologous fibrin biopolymer; HF_L_—defect filled by heterologous fibrin biopolymer and photobiomodulation; PHF—defect filled by deproteinized bovine bone particles incorporated into fibrin biopolymer; PHF_L_—defect filled by deproteinized bovine bone particles incorporated into fibrin biopolymer and photobiomodulation. (**D**) PBMT—transoperative laser photobiomodulation treatment (Dosimetry)—continuous mode, infrared spectrum, 0.028 cm^2^ beam area, 808 nm wavelength, 100 mW power, 3.57 W/cm^2^ target irradiance, energy density of 214.29 J/cm^2^ per point, 60 s/point, application at five points of the defect surface clockwise and central point (single session). Each point received an energy dose of 6 J for 60 s/point, and the total energy area of 30 J. (**E**) Experimental periods: Half of each experimental group was euthanized in 14 days and the other half in 42 days.

Sixty rats were obtained from the bioterium of the Ribeirão Preto (University of São Paulo—USP, Brazil), following the inclusion criteria: adults (*Rattus norvegicus*), Wistar hannover strain, healthy males, age 90 days, and weighing of approximately 320 g (Figure 9A).

The animals were received at the age of 42 days and during the experimental period they were kept in conventional cages containing initially four animals each (change according to the animal’s weight), with feeders and drinkers “ad libitum”, irradiated feed—Nuvilab rodents (Nuvilab^TM^ rat chow, Nuvital, Colombo, Brazil) and filtered water, in an acclimatized environment, air exhaust, light-dark period 12L/12D, temperature 22 ± 2 °C, humidity 60 ± 10%, lighting 150 lux/1 m floor, max noise 70 DCb.

This study was approved by the Ethics Committee on the Use of Animals (CEUA) of the Bauru Dental School—University of São Paulo (FOB-USP), CEUA-Proc. No 005/2020. The present study strictly followed the ARRIVE (Animal Research: Report of in vivo Experiments) checklist in order to allow researchers to properly examine the work, assess its methodological rigor and reproduce the methods and results [81,82]. During the entire experiment, the animals were monitored for pain expression, by observing whether the animal was apathetic, depressed, aggressive or hyper-excited, mainly due to such traits that are variables of its usual behavior. It was also controlled whether there were changes in walking, posture or facial expression, water and food consumption, in addition to clinical symptoms.

The animals were randomly distributed into 5 groups (*n* = 12) according to the type of defect filling and photobiomodulation treatment (Figure 9C–E): BC_L_—defect filled by blood clot and laser photobiomodulation therapy; HF—defect filled by heterologous fibrin biopolymer; HF_L_—defect filled by heterologous fibrin biopolymer and laser photobiomodulation therapy; PHF—defect filled by deproteinized bovine bone particles incorporated into heterologous fibrin biopolymer; PHF_L_—defect filled by deproteinized bovine bone particles incorporated into heterologous fibrin biopolymer and laser photobiomodulation therapy.

### 4.4. Experimental Procedure

Surgical procedures were standardized and performed by the same team of professionals. The animals were submitted to intraperitoneal general anesthesia in the left lower abdominal quadrant, using the sedative ketamine hydrochloride 80 mg/kg of animal weight (Dopalen^TM^, Sespo Industria e Comercio Ltd., São Paulo, Brazil) and the muscle relaxant xylazine hydrochloride 10mg/kg of animal weight (Anasedan^TM^, Sespo Industria e Comercio Ltd., São Paulo, Brazil), with strict monitoring.

Then, trichotomy was performed with the aid of a hair trimmer (Philips^TM^ Multigroom QG3250, São Paulo, Brazil) in the frontal–parietal bone region, between the external auricular pavilions and weighed on an analytical balance (MicroNal^TM^ Precision Equipment, São Paulo, Brazil).

Antisepsis of the shaved region, including the fur around this area, was performed with a 10% topical solution of Polyvinyl Pyrrolidone Iodine PVPI (Povidine^TM^, Vic Pharma Ind e Comercio Ltd., São Paulo, Brazil).

The surgical procedure took place independently, on a covered bench, on a wooden table covered with cork, with material exchange for each specimen. The animals were fixed to the operating table, positioned in the prone position. Then, a 4 cm semilunar incision was made with a No. 15 carbon steel scalpel blade (Embramax^TM^, São Paulo, Brazil) in the integument and the periosteum was carefully detached with the aid of the syndesmotome and folded back together with the other tissues, exposing the surface exterior of the parietal bones.

A circular osteotomy of 8.0 mm in diameter was performed in the center of the parietal bones with the aid of a trephine drill (Neodent^TM^, Curitiba, Brazil) adapted to the contra-angle (Driller^TM^, São Paulo, Brazil) coupled to an electric micromotor (Driller^TM^ BLM 600 Baby, São Paulo, Brazil), at low speed (1500 rpm), under constant and abundant sterile saline solution 0.9% sodium chloride JP^TM^ (JP Farma—Pharmaceutical Industry, Ribeirão Preto, Brazil) to avoid bone necrosis by thermal action, thus obtaining a rounded bone fragment, without spicules, preserving the integrity of the dura mater and the brain (Figure 9B).

In the animals of the BC_L_ group, the defects were performed and not filled by biomaterials (blood clot only). In the animals of Groups HF and HF_L_, the defects were filled by the fibrin biopolymer. In PHF and PHF_L_ groups, the defects were filled with deproteinized bovine bone particles incorporated into the fibrin biopolymer.

The biomaterial was previously weighed on an analytical balance (MicroNal^TM^ Precision Equipment, São Paulo, Brazil) in order to completely fill the surgical cavity. After complete polymerization of the biopolymer with the bone matrix, the resulting compound was transferred to the defect site without exerting pressure on the brain.

Subsequently, the experimental groups BC_L_, HF_L_ and PHF_L_ were submitted to laser photobiomodulation treatment. With the surgical cavity still exposed, the laser was positioned perpendicularly at five points on the surface of the defect in a clockwise direction (12 h, 3 h, 6 h, 9 h), in addition to a central point in a single session. (Figure 9C).

The tissues of the surgical area were repositioned, taking care that the periosteum covers the cavities, and then the integument was sutured (simple stitches) with 4–0 silk thread (Ethicon^TM^, Johnson and Johnson Company, São Paulo, Brazil). The region was carefully cleaned with gauze moistened with topical antiseptic, 2% chlorhexidine (Riohex^TM^ Pharmaceuticals Rioquimica, São José do Rio Preto, Brazil).

The animals were placed in the lateral decubitus position in cages and exposed to incandescent light for complete anesthetic recovery. Immediately after the surgical procedures, the animals received a single dose of the antibiotic Flotril^TM^ 2.5% (Schering-Plough, Rio de Janeiro, Brazil), at a dose of 0.2 mL/kg and the analgesic Dipirona Analgex V^TM^ (Agener União, São Paulo, Brazil) at a dose of 0.06 mL/kg, in intramuscular applications. Analgesic application was maintained for 3 days, in addition to continuity with the analgesic Acetaminophen (Paracetamol, Generic medication, Medley, São Paulo, Brazil) at a dose of 200 mg/kg, 6drops/animal dissolved in the water available in the drinking fountain until the period of euthanasia.

### 4.5. Laser Photobiomodulation Therapy Protocol

Groups BC_L_, HF_L_ and PHF_L_ were submitted to treatment with Therapy XT DMC^TM^ (São Carlos, Brazil), in continuous mode, infrared spectrum, with active medium GaAlAs (Gallium-Aluminum-Arsenide), beam area of 0.028 cm^2^, wavelength of 808 nm, output power 100 mW, target irradiance 0.6m W/cm^2^, energy density 210 J/cm^2^ per spot, 60 s/spot, application to five points of the defect surface clockwise (12 h, 3 h, 6 h, 9 h), plus a central point (single session). Each point received an energy dose of 6 J for 60 s/point, and the area received a total energy of 30 J. Only one application was performed transoperatively [10] (Figure 9D) (Figure S1, Supplementary Materials).

### 4.6. Euthanasia and Tissue Collection

After the periods of 14 and 42 days after surgery, 6 animals from each group per period were weighed and euthanized by the general anesthetic overdose method (triple dose—240 mg/kg ketamine + 30 mg/kg xylazine). Then, the animals returned to the box, as they remained in stage II (excitation) for a longer time.

After confirming the death of the animal, the defect region of each animal was carefully removed with the aid of straight surgical scissors, preserving the supraperiosteal soft tissues and fixed in 10% phosphate-buffered formalin (Allkimia^TM^—Commerce of Materials for Laboratories Ltd., Campinas, Brazil), pH 7.2 for 24 h, and later destined to the examination in the microtomograph.

### 4.7. X-ray Computed Microtomography (µ-CT)

After fixation of the bone fragments, the pieces were submitted to an X-ray beam scanning on the SkyScan 1174v2 computerized microtomograph (Bruker-microCT, Kontich, Belgium). The X-ray beam sources (Cone-Beam) were operated at 50 kV, 800 uA, using a Cu+Al filter. The pieces were packed in tubes, positioned and fixed in the appropriate sample holder for the equipment, with a useful wax, enabling stabilization, in order to prevent any type of movement during scanning. Then, they were rotated 180°, with a “rotation step” of 0.7, and a spatial resolution of 19.78 µm pixel size (1024 rolls × 1304 columns), generating an acquisition time of 41 min and 33 min and 25 s/sample.

The images of each specimen were analyzed and reconstituted with the specific software 64 Bits 270013 (Bruker^TM^, Kontich, Belgium) and the NRecon^TM^ Program (version.1.6.8.0, SkyScan, 2011, Bruker-microCT, Kontich, Belgium) in about 1000 to 1100 slices according to the parameters adopted anatomical. Data Viewer^TM^ version 1.4.4 64bit software (linear measurements of coronal, transaxial and sagittal axes) and CTvox^TM^ version 2.4.0r868 (Bruker MicroCT, Kontich, Belgium) were used for the two-dimensional and three-dimensional visualization, respectively, followed by qualitative and quantitative analysis of newly formed bone tissue [25].

Morphometric quantifications were determined using the images (coronal position) and the region of defect or interest (ROI), performed manually on all images every ten times. After selecting the volume of interest (VOI), binarization was performed, enabling the distinction between the grafted material and the newly formed bone. Thus, to perform the 3D analysis, adequate threshold ranges were determined for the biomaterial (130–255), and the newly formed bone (130–72). Soft tissue are hypodenses and the data total soft tissue volume (StV) and percentage of soft tissue (StV/TV) were obtained from the difference between StV = TV—(Biomaterial Volume BioV + New bone Volume NbV) and the percentage StV/TV = 100—(BioV/TV + NbV/TV).

### 4.8. Histotechnical Processing

After Collecting the microtomographic images, the specimens were washed in running water for 24 h and subjected to demineralization in ethylenediaminetetraacetic acidan (EDTA) solution, a solution containing 4.13% tritiplex^TM^ III (Merck KGaA, Hessen, Germany) and 0.44% sodium hydroxide. sodium (Labsynth^TM^, São Paulo, Brazil) with weekly changes of the solution for an approximate period of 60 days. During these EDTA exchange intervals, radiographic analyses were performed with Insight Adult IP-21 F-Speed—Carestream^TM^ periapical film (Carestream Health, New York, USA) to confirm the demineralization process. After complete demineralization, the pieces were dehydrated in an increasing series of ethyl alcohol, diaphanized in xylene and embedded in Histosec^TM^ processed paraffin (Merck, Hessen, Germany).

Subsequently, semi-serial coronal sections were performed considering the central region of the defect with the aid of a Leica^TM^ RM2245 semiautomatic microtome (Leica Biosystems, Wetzlar, Germany), with a thickness of 5 µm for hematoxylin-eosin (HE) staining, Masson’s trichrome (MT), and Picrosirius-red (PRS).

### 4.9. Histomorphometric Analysis of HE-Stained Defects

For the histomorphological description of the areas of the bone defect, in all specimens, the entire extension of the defect was considered, to evaluate the pattern of bone repair in all groups. Thus, it was possible to analyze in each defect the presence of granulation tissue, inflammatory infiltrate, the presence and quality of immature or mature/lamellar bone and the degree of filling of the neoformed tissue in HE (4×) and MT (4× and 40×).

For this, 4 semi-serial sections of the surgical bed of each defect were evaluated in an Olympus^TM^ BX50 light microscope (Olympus Corporation, Tokyo, Japan) and the photographs were captured in 4× and 40× objectives, HE staining and Masson’s trichrome with a digital camera attached. (Olympus DP 71, Tokyo, Japan) using image capture software DP Controller 3.2.1.276 (2001–2006, Olympus Corporation, Tokyo, Japan) with image size specifications 4080 × 3072 pixels and spot 0.1%

Volume density (VVi) is defined as the volume fraction occupied by a given constituent (graft, inflammatory infiltrate, connective tissue, bone tissue and bone marrow) of the whole (graft defect + reaction tissue) and can be obtained in histological sections as area fraction, i.e., VVi = AAi. After capturing the images covering the entire defect using a 4x objective, HE staining and storing in Tag Image File Format (TIFF), the entire defect was reconstructed in Adobe Photoshop CS6 (Adobe Systems, San Jose, CA, USA). Then, the entire defect was evaluated in the image analysis program AxioVision, where the total analyzed area (A) and the area occupied by each constituent in the defect (Ai) were determined by the PIXEL measurement unit. The volume density (Vvi) of each type of structure was calculated by the relationship: Vvi = AAi = Ai/A.100 [83].

### 4.10. Birefringence Analysis of Collagen Content of Bone Defects

The PRS-stained sections were evaluated under polarized light to determine the quality and quantity of the newly formed organic matrix over the experimental defect healing periods. Defect images were obtained using a Leica DFC 310FX high resolution digital camera (Leica^TM^, Microsystems, Wetzlar, Germany) connected to a Leica DM IRBE confocal laser microscope and LAS 4.0.0 capture system (Leica^TM^, Microsystems, Heerbrugg, Switzerland).

To allow for the identification and analysis of collagen quantity and quality by the birefringence of fiber bundles organization, the central fields of the defects were analyzed under a polarized light microscope with 10x magnification. Three histological fields were captured corresponding to the full extent of the defect. All remaining bones and dark areas (without tissue/material) present in the histological fields were removed to avoid counting these fibers and/or regions in Adobe Photoshop CS6 software (Adobe Systems, San Jose, CA, USA).

Images were transferred to AxioVision Rel imaging software 4.8 (Carl Zeiss MicroImaging GmbH, Jena, Germany) and total area was determined with dashed lines, area of biomaterial particles, connective tissue area and newly formed tissue area, yielding values in Pixels^2^.

Using the interactive Processing-Segmentation-Thershold tool, the RGB (Red, Green e Blue) color standard was determined for each color. Then, the analysis of the density area or the percentage (%) of each type of fiber by color was evaluated. Tissue bone was recognized by its random, disorganized fibrillar pattern, usually with polarization colors ranging from bright green/yellow (poorly organized bone) to orange–red (lamellar bone), depending on fiber thickness and organization. (Figure S2, Supplementary Materials).

### 4.11. Statistical Analysis

The volumetric quantitative data obtained in the morphometric evaluations of the microtomography, the area and percentages obtained by the histomorphometry in the sections stained in HE and PRS were submitted to the Kolmogorov–Smirnov normality test. To assess the presence of statistical difference between the periods (14 and 42 days) for each study group, the unpaired Student’s “*t*” test was applied. In order to verify the differences between the groups (BC_L_, HF, HF_L_, PHF and PHF_L_) in each experimental period, we applied the Analysis of Variance (ANOVA) test to one criterion followed by Tukey’s post-test. The tests were performed by the GraphPad Prisma software 5 (GraphPad Software Inc., San Diego, CA, USA) adopting a significance level of 5% (*p* < 0.05) for all parameters.

## 5. Conclusions

The results of the present study showed that the transoperative protocol of laser photobiomodulation in critical bone defects in the calvaria of rats filled with deproteinized bovine bone particles associated with heterologous fibrin biopolymer with alteration in the proportionality of fibrinogen were relevant in all evaluation parameters, (BV, 9.94 ± 1.51 mm^3^; BA/TA, 25.71 ± 2.79%, red/thicker fibers, mean of 85.03 ± 5.54% at 42 days), showing promise in the bone repair process.

These findings encourage prospective studies in order to investigate and explore the effects of low-level laser on other graft associations with the proportionality of fibrin, proposed in this study. In addition, a more accurate study evaluating the best fibrinogen concentration should be performed in the future to standardize the ideal heterologous fibrin scaffold.

## Figures and Tables

**Table 1:** Selection of laser processing parameters that allow their correlation with desired characteristics.

Laser Processing Parameters	Measurement/Unit	Explanation of Parameter Selection
Continuous mode		Avoids 6% energy loss by reflection compared to off contact mode [36]
Infrared spectrum		Depth of penetration, >absorptivity by cytochrome C oxidase—bone [37]
GaAlAs		37% intensity loss after crossing 2 mm depth. Precalvarial tissue thickness in the rat 0.56 mm. minimum loss [38]
Beam área	0.028 cm^2^	
Wavelength	808 nm	High wavelengths are more resistant to dispersion than lower ones; penetrate deeply into tissue; low water chromophore interference [39]
Output power	100 mW	Energy without microthermal tissue damage (>500 mW) [40]
Target irradiance (I)	3.57 W/cm^2^	Calculated as: I = potency (W)/beam area (cm^2^). Biphasic response: “Arndt-Schulz Law”—weak stimuli accelerate slightly, stronger stimuli increase even more until it reaches a peak; even stronger stimuli suppression [39]
Energy density (E)	214.29 J/cm^2^ per spot	Calculated as: E = Dose energy (J)/beam área (cm^2^) photostimulatory effects = 1–10 J/cm^2^; photoinhibitory effects ≥10 J/cm^2^) [41]
Time	60 s/spot	
IntraoperativeApplication	Five points of the defect surface clockwise (12 h, 3 h, 6 h, 9 h), plus a central point (single session).	To treat the entire injured area considering the radiation loss by scattering and reflection; >effect on cells—early stages of repair—>cell proliferation and division—>volume of newly formed bone [38,42]
Energy dose (D)	6 J for 60 s/point	Calculated as: D = potency (W) × point time (s). 37% scattering loss ≥ 2 mm depth (0.56 mm overlying soft tissue) [43,44]

## Data Availability

The data presented in this study are available on request from the corresponding author. The data are not publicly available due to they are part of a post-doctoral report not yet deposited in a public repository.

## Supplementary Materials

The following supporting information can be downloaded at: https://www.mdpi.com/article/10.3390/molecules28010407/s1, Figure S1: Materials used: (A) Deproteinized bovine matrix (Ministry of Health Registration No. 806.969.30002); (B) Fibrin biopolymer purified from snake venom (Ministry of Health Registration No. 1020140114327 and No. 1020140114360); (C) Therapeutic Laser, Therapy XT DMC^®^ (Ministry of Health Registration 80030810157), Figure S2: RGB—green-yellow-red for interpretation of color references in the birefringence analysis of collagen fibers, used in this study.

## Sample Availability

Samples of the Bio-Oss^TM^ matrix and Heterologous Fibrin Biopolymer are available from the authors.
