# Supplementary material for: Tissue Bioengineering with Fibrin Scaffolds and Deproteinized Bone Matrix Associated or Not with the Transoperative Laser Photobiomodulation Protocol
Source: Molecules. 2023 Jan 3;28(1):407. doi: 10.3390/molecules28010407 (PMC9824823; doi:10.3390/molecules28010407)
Supplement: Supplementary file 1 [file molecules-28-00407-s001.zip › molecules-2000396-supplementary.pdf]

Supplementary Materials

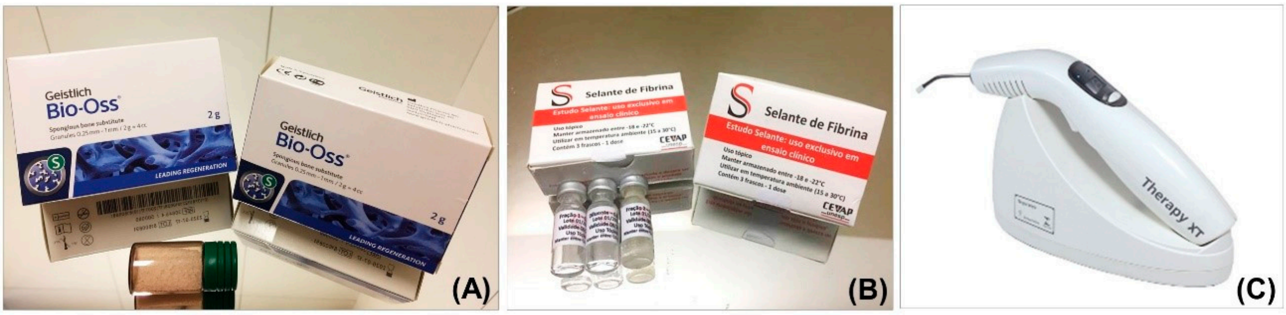

Figure S1: Materials used: (**A**) Deproteinized bovine matrix (Ministry of Health Registration No. 806.969.30002); (**B**) Fibrin biopolymer purified from snake venom (Ministry of Health Registration No. 1020140114327 and No. 1020140114360); (**C**) Therapeutic Laser, Therapy XT DMC® (Ministry of Health Registration 80030810157)

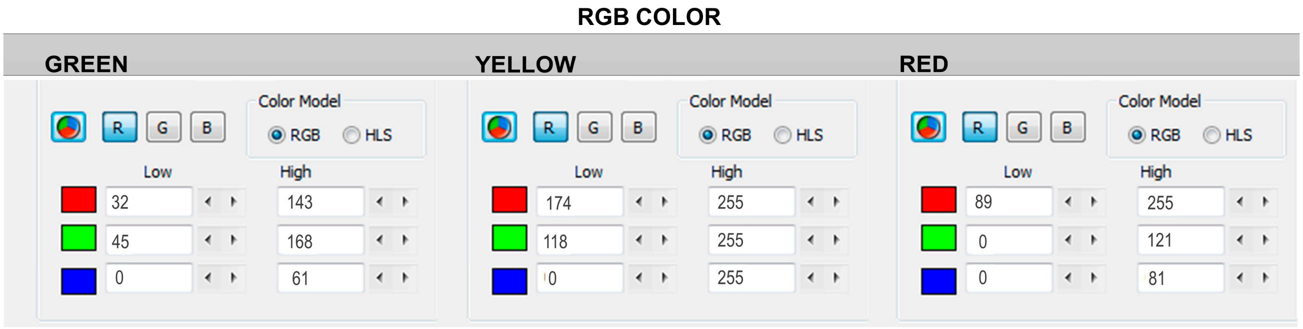

Figure S2. RGB—green-yellow-red for interpretation of color references in the birefringence analysis of collagen fibers, used in this study.
